# Applying a cost-based pricing model for innovative cancer treatments subject to indication expansion: A case study for pembrolizumab and daratumumab

**DOI:** 10.1371/journal.pone.0293264

**Published:** 2024-02-01

**Authors:** R. J. S. D. Heine, F. W. Thielen, R. H. J. Mathijssen, R. W. F. van Leeuwen, M. G. Franken, C. A. Uyl-de Groot

**Affiliations:** 1 Erasmus School of Health Policy and Management (ESHPM), Erasmus University Rotterdam, Rotterdam, The Netherlands; 2 Erasmus Centre for Health Economics Rotterdam (EsCHER), Erasmus University Rotterdam, Rotterdam, The Netherlands; 3 Department of Medical Oncology, Erasmus Medical Center Cancer Institute, Rotterdam, Rotterdam, The Netherlands; 4 Department of Hospital Pharmacy, Erasmus University Medical Center, Rotterdam, Rotterdam, The Netherlands; Kings College Hospital, UNITED KINGDOM

## Abstract

**Background:**

Expanding the indication of already approved immuno-oncology drugs presents treatment opportunities for patients but also strains healthcare systems. Cost-based pricing models are discussed as a possibility for cost containment. This study focuses on two drugs, pembrolizumab (Keytruda) and daratumumab (Darzalex), to explore the potential effect of indication broadening on the estimated price when using the cost-based pricing (CBP) model proposed by Uyl-de Groot and Löwenberg (2018).

**Methods:**

The model was used to calculate cumulative yearly prices, cumulative prices per indication, and non-cumulative indication-based prices using inputs such as research and development (R&D) costs, manufacturing costs, eligible patient population, and a profit margin. A deterministic stepwise analysis and scenario analysis were conducted to examine how sensitive the estimated price is to the different input assumptions.

**Results:**

The yearly cumulative cost-based prices (CBPs) ranged from €52 to €885 for pembrolizumab per vial and €823 to €31,941 for daratumumab per vial. Prices were higher in initial years or indications due to smaller patient populations, decreased over time or after additional indications. Sensitivity analysis showed that the number of eligible patients had the most significant impact on the estimated price. In the scenario analysis the profit margin contributed most to a higher CBPs for both drugs. Lower estimates resulted from assumed lower R&D costs.

**Discussion:**

The estimated CBPs are consistently lower than Dutch list prices for pembrolizumab (€2,861), mainly resulting from larger patient populations in registered indications. However, daratumumab’s list prices fall within the range of modeled CBPs depending on the year or indication (€4,766). Both CBPs decrease over time or with additional indications. The number of eligible patients and initial R&D costs have the most significant influence on the CBPs. These findings contribute to the ongoing discussions on pharmaceutical pricing, especially concerning cancer drugs with expanding indications.

## Introduction

The International Agency for Research on Cancer (IARC) reported an estimated 19.3 million new cases of cancer in 2020 [1], forecasting a 47% surge in its incidence between 2020 and 2040 [2]. In several countries cancer has become the leading cause of death, surpassing cardiovascular diseases [3]. Rising cancer rates are paralleled by an increase in cancer drug development, especially in immuno-oncology (IO) [4,5].

The new therapies that made it to the market have, however, driven up the spending on drugs both in the European Union (EU) and the United States (US) [6,7]. In the EU-28, the estimated gross sales of oncology drugs grew from €18.0 billion in 2012 to €30.2 billion in 2017, an annual growth rate of 11% [7]. During the same period, however, the sales volume of oncology drugs only rose by 2% per year. The increment in spending was, therefore, primarily driven by the introduction of new high-priced therapies [7]. A study in the US estimated that if all eligible patients would have access to all new drugs or drug indications that were approved in 2018, the extra spending would be $39.5 billion, representing an increase over 75% compared to 2017 expenditures [8].

DeMartino et al. (2021) identified 46 new oncology drug approvals in 2018 of which 29 (63%) were approvals of the same drug for new indications. Therefore, the expansion of indications for existing oncology drugs now surpasses the number of approvals for new oncology drugs. Pembrolizumab (Keytruda^®^) and Daratumumab (Darzalex^®^) are two examples of antineoplastic agents with high drug costs [9,10]. Pembrolizumab, a programmed death receptor (PD-1) inhibitor, was firstly approved in September 2014 for unresectable or metastatic melanoma by the Food and Drug Administration (FDA) [11] and in July 2015 by EMA [12]. Since its initial approval, pembrolizumab has been granted 37 new FDA approvals across various indications. [12] Daratumumab (Darzalex^®^) received approval from the FDA in November 2015 and in May 2016 from the EMA [13], for patients with multiple myeloma (MM). Daratumumab has been granted 9 new indications by the FDA since first approval.

Moreover, both pembrolizumab and daratumumab rank in the top five for increased drug spending between 2017 and 2018 in the Netherlands: based on list prices, spending rose 123.8% for pembrolizumab and 171.4% for daratumumab [14].

In response to soaring drug prices, Uyl-de Groot and Löwenberg (2018) proposed a cost-based pricing model [9]. Although their model considers expenditures related to research and development (R&D), costs for drug manufacturing, sales and marketing, the potential patient population within the remaining patent period, and a profit margin, it does, however, not take broadening expanding of indications into account. The model by Uyl-de Groot and Löwenberg (2018) was previously used to calculate prices for Cell and Gene therapies (CGTs) [10], but it was not, however, yet used for (expensive) anticancer drugs with expanding indications. Other measures such as minimum effectiveness criteria, managed entry agreements (MEAs), multi decision analyses (MCDA) and differential/tiered pricing including indication-based pricing are being utilized to address rising spending [15]. However, in this study we focus on another measure namely, transparent pricing models.

Cost-based models can contribute to the future development of sustainable pricing models and the debate on “fair” drug pricing. Therefore, we advanced the model of Uyl-de Groot and Löwenberg (2018) [9] by incorporating the element of broadening of indications. Daratumumab and pembrolizumab represent both histology-agnostic and histology specific, drugs that are subject to indication broadening. We modelled a range of cost-based prices for both pembrolizumab and daratumumab and compared these with known list prices.

## Materials and methods

### Pricing model

The original model of Uyl-de Groot and Löwenberg (2018) [9] incorporates research and development costs (C_rd_), manufacturing and marketing costs (C_man_), the eligible patient population over the remaining patent time (N_p_), and a profit margin (M_p_) and [9] estimates a cost-based price (CBP) per treatment (C_tx,_ see Eq 1).


$$C_{tx}=\left(\frac{C_{rd}}{N_{p}}+C_{man}\right)*(1+M_{p}).$$ (Eq 1)

Our algorithm (see Eq 2) advances the model of Uyl-de Groot and Löwenberg (2018) [9] by including expanding indications. The CBP is calculated for each time interval in years (C_tx(i)_) by summing the initial cost of research and development (C_rd_) and the number of new indications (N_ind_) multiplied by the cost of research and development for a new indication (C_ex_). The total R&D costs are divided by the sum of eligible patients over the remaining patent time (N_p_). Costs for manufacturing are derived from the weighted dose (D_ind_) multiplied by the cost of manufacturing per gram (C_man_). Lastly, the profit margin (M_p_) and sales & marketing margin (M_sm_) are applied. For this study, we also adjusted the algorithm to calculate an estimated CBP per vial facilitating a direct comparison with current prices.


$$C_{tx(i)}=\left(\frac{{\sum_{i=t1}^{n}C}_{rd}{+(N}_{ind}*C_{ex})}{\sum_{i=t1}^{n}\;N_{p}}+\sum_{i=t1}^{n}D_{ind}\;C_{man}\right)*(1+M_{p}+M_{sm})\;\;$$ (Eq 2)

We used the population *“more developed regions”* as defined by the *United Nations* (UN-MDR), which includes Europe, North America, Australia/New Zealand, and Japan [16].

### Costs for research and development (C_rd_) and cost for a new indication (C_ex_)

Estimating initial and new indication R&D costs was not feasible due to a lack of stratified reporting of these expenses by type of product or indication from the pharmaceutical companies Janssen Pharmaceutica and Merck. Therefore, we used the mean estimation for antineoplastic and immunomodulating agents from Wouters et al. (2020) [17] as a base-case namely €4937.4 million (95% CI, €3446.4 million–€6641.9 million) (see Table 1). This accounts for the costs of failed trials, cost of capital (10.5%) and all clinical phases of the drug development.

**Table 1:** Input parameters for the base-case analysis.

*Category.*	*Description*	*Value*	*References*
*Cost*	R&D costs associated with the development of antineoplastic and immunomodulating agents, capitalized and risk adjusted. Unadjusted for inflation and currency change.	€4937.4 million	Wouters et al. (2020) [17]
	Cost of manufacturing per gram of mAbs	€55	Ou Yang et al. (2019) [24]
	R&D costs associated with each new indication.	€347 million	Nosengo (2016) [18]
*Incidence rate per 100,000*	Multiple myeloma	7.6	IARC [1]
	Melanoma	21.7	IARC [1]
	Lung Cancer	69.5	IARC [1]
	Head and neck cancer	21.8	IARC [1]
	Hodgkin Lymphoma	2.5	IARC [1]
	Bladder cancer	26.3	IARC [1]
	Gastric and Oesophagus cancer	32.1	IARC [1]
	Cervical cancer	13.3	IARC [1]
	Non-Hodgkin Lymphoma	19.2	IARC [1]
	Liver cancer	14.4	IARC [1]
	Non melanoma skin cancer	80.3	IARC [1]
	Kidney cancer	19.3	IARC [1]
	Oesophagus cancers	8.0	IARC [1]
	Corpus uteri cancer	33.7	IARC [1]
	Colorectum cancer	68.1	IARC [1]
	Breast cancer	142.0	IARC [1]
*Patent expiry (year)*	Daratumumab	2025	Busse & Lüftner (2019) [21]
	Pembrolizumab	2028	Busse & Lüftner (2019) [21]
*Profit margin (in %)*	Profit margin	20%	Uyl-de Groot & Löwenberg (2018) [9]

mAbs: Monoclonal antibodies.

Literature on R&D costs for broadening indications is also lacking. However, some studies published costs associated with repurposing of existing drugs. Although these costs might differ from seeking a new indication, repurposing costs were considered the closest approximation to the incurred investment needed for broadening indications. Nosengo (2016) [18] estimated the costs associated with the repurposing of a new drug to be €347 million due to the possibility to skip phase I trials and the lower risk of serious side effects.

### The eligible patient population during the remaining patent period

Incidence rates from the International Agency for Research on Cancer (IARC) [1] were used to discern the eligible patient population for both pembrolizumab and daratumumab in all relevant indications across selected countries (UN-MDR). This was achieved by multiplying non-country-specific incidence rates with the estimated population from the 2022 *UN Revision of World Population Prospects [16],* retrieved from the R package wpp2022 [19].

### Eligible patient populations

The obtained patient populations were adjusted to account for cancer subtype, stage, treatment line, symptomatic disease (only in case of daratumumab), and market share. We also accounted for patients not treated (assumed to be 25% for pembrolizumab and 0% or 5% for daratumumab), patients participating in clinical trials (assumed to be 10%) and percentage of eligible patients, informed through clinical opinion [20]. Lastly, market share (MS) was defined as the existence of a competitor in the market (no competitor for daratumumab and two competitors for pembrolizumab (i.e., nivolumab (Opdivo®) monotherapy and nivolumab plus ipilimumab (Yervoy®) combination therapy for certain indications. The MS for pembrolizumab was, therefore, restricted to 50% in case one competitor was approved and 33% if both competitors were approved.

### Patent expiry prediction

Patent expiry was predicted for pembrolizumab and daratumumab in 2028 and 2025, respectively [21]. The selected patent expiry year is conservative for pembrolizumab as the patent for the United States (US) is predicted to expire in 2036. Moreover, Janssen Pharmaceutica has developed a subcutaneous injection formulation that will likely remain under patent protection until 2035 [22]. This should, however, not hamper the introduction of generics, and probably only plays a role in MS retention.

A key deviation from the original model by Uyl-de Groot and Löwenberg (2018) [9] is the time-dependency to enable the inclusion of broadening indications. (i.e., the remaining patent years are dependent on the time within the model).

### Cost of pharmaceutical manufacturing

Manufacturing costs for both drugs were not found in the literature. However, the production and advances made with regard to bioprocessing in the production of monoclonal antibodies (mAbs) have been studied and in some instances costs associated with their production have been reported [23–25]. Despite the variance in production cost estimates (€34 - €174) the mean estimated cost per gram of a mAbs ranged between €55 and- €68 we used €55 per gram for the base-case. In our model, cost of manufacturing are used in conjunction with the dose per gram of mAbs (Appendix V & VI).

### Adjusting for inflation and currency change

All prices and costs were expressed in 2022 Euro (EUR). When necessary, prices and cost were adjusted for inflation and currency exchange rates, following the methodology of Turner et al. (2019) [26]. We assumed all cost inputs to be non-tradable resources.

### Profit margin

The model proposed by Uyl-de Groot and Löwenberg (2018) recommended a variable profit margin linked to the clinical benefit [9], ranging from 20% for marginal benefits to 40% for high-level of benefits. However, using different profit margins based on clinical benefit for each indication expansion would overcomplicate the calculations. Therefore, we assumed a 20% profit margin for the base-case and varied the profit margin in sensitivity analyses.

### Current value-based prices

An 1,800 mg daratumumab subcutaneous solution is listed for €8,735 in the US [27] and €4,766 in the Netherlands [28]. The list price for pembrolizumab varies across countries ranging from €5,350 in the US [29], €3,078 in the United Kingdom (UK) [30], and €2,861 in the Netherlands [31] for a 4ml vial containing 25mg/ml.

### Sensitivity analysis

To address uncertainty in model input parameters, we performed a stepwise deterministic one-way sensitivity analysis (DSA), varying cost inputs, profit margin and the number of eligible patients. Prices for both drugs were recalculated with increments of 10%, ranging from -30% to +30%.

We further conducted scenario analysis for inputs where lower and upper bounds were available in the literature, keeping all other inputs constant (see Table 2). The applications of different scenarios provided a range of CBPs around the base-case. This is valuable considering the lack of exact inputs and dependence on surrogate inputs to populate the model. Costs of initial R&D were retrieved from Wouters et al. (2020) [17] which reported a lower bound of €3,446.4 million and an upper bound of €6,641.9 million for antineoplastic and immunomodulating agents. Moreover, a scenario was modelled with R&D costs that were not capitalized, as the inclusion of cost of capital and the rate at which it should be applied are still under debate [32]. For costs associated with each new indication, we created a scenario following the guidelines set out by AIM, namely 10% of initial R&D costs (€4,937.4 million) [33]. A lower bound of €33 and an upper bound of €174 informed by Kelly (2009) [25] was used for manufacturing costs. Two scenarios modelled the profit margin: a profit margin of 0% and a profit margin of 76.5% (i.e., corresponding to the highest estimate found in Ledley et al. (2020) [34]. One scenario modelled a less conservative remaining patent period, namely 2035 for daratumumab and 2036 for pembrolizumab. Lastly, one scenario modelled implementing indication-based price (IBP) as certain countries within the EU have implemented IBP [35]. The indication-based cost-based price (IBCBP) was calculated for each new indication, the price reflects non-cumulative R&D costs and patient population.

**Table 2:** Inputs utilized in base-case and scenario analysis for daratumumab and pembrolizumab, both unadjusted and adjusted for PPP and inflation.

*Input*	*Unadjusted input*	*Adjusted for 1^st^ year daratumumab (2015)*	*Adjusted for 1^st^ year pembrolizumab (2014)*
***Costs associated with R&D***			
*Base-case*	$4,461,200,000 [17]	€3,795,249,282	€3,165,890,999
*Scenario 1 Cost R&D (CI)*	$3,114,000,000 [17]	€2,649,154,098	€2,209,850,393
*Scenario 2 Cost R&D (CI)*	$6,001,300,000 [17]	€5,105,449,097	€4,258,823,109
*Scenario 3 Cost R&D (uncapitalized)*	$1,032,000,000 [17]	€877,947,023	€732,358,897
***Cost associated with each new indication***			
*Base-case*	$300,000,000 [18]	€267,020,244	€222,740,834
*Scenario 4 based on AIM method*	$446,120,000 [33]	€379,524,928	€316,589,100
***Cost of product manufacturing (per gram)***			
*Base-case*	$51 [24]	€42.61	€35.55
*Scenario 5 cost manufacturing (low)*	$26 [25]	€25.89	€21.59
*Scenario 6 cost manufacturing (high)*	$134 [25]	€133.43	€111.30
***Profit margin in %***			
*Base-case*	20 [9]	20	20
*Scenario 7 profit margin 0%*	0	0	0
*Scenario 8 highest profit margin*	76.5 [34]	76.5	76.5
***Patent period***			
*Scenario 9 patent period*	2035 & 2036[22]	2035	2036
***Type of cost-based price model***			
*Scenario 10 indication-based pricing (IBP)*	IBP	IBP	IBP

## Results

**Fig 1:**
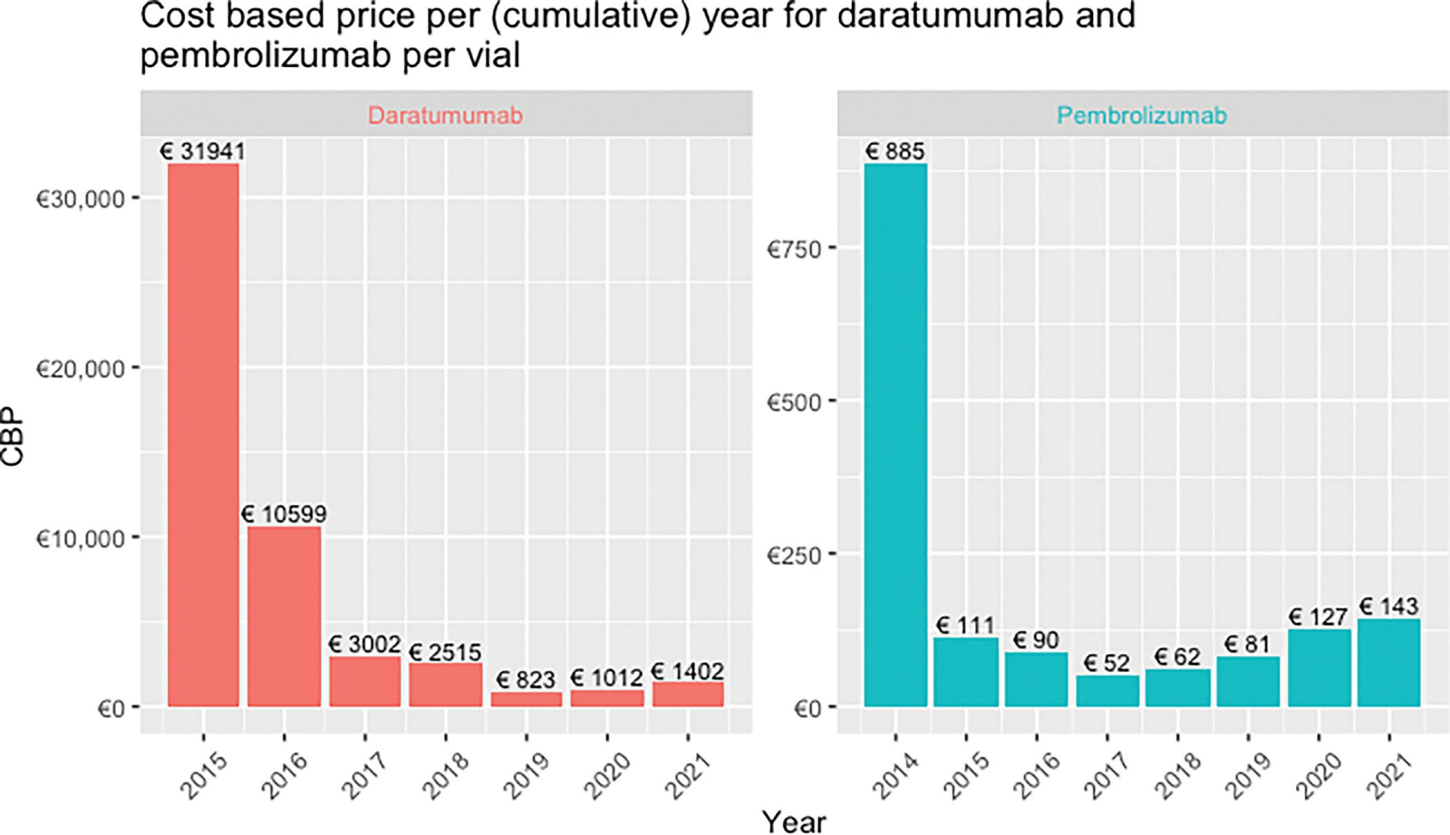
CBP per cumulative year for daratumumab and pembrolizumab per vial.

Fig 1 shows the CBP per year for both drugs. The introduction year (i.e., the first year a drug accesses the market), both cost-based prices are considerably higher due to the initial R&D costs and the relatively small initial patient populations, namely €885 for pembrolizumab and €31,941 for daratumumab. In the years thereafter, the CBPs decreased considerably; the minimum of prices amounted to €52 (pembrolizumab) and to €823 (daratumumab). The CBPs initially decreased considerably but slowly increased after four to five years, partly due to the diminishing patent period (i.e., in case the remaining patent period becomes smaller the time remaining to recoup subsequent investments decreases).

**Fig 2:**
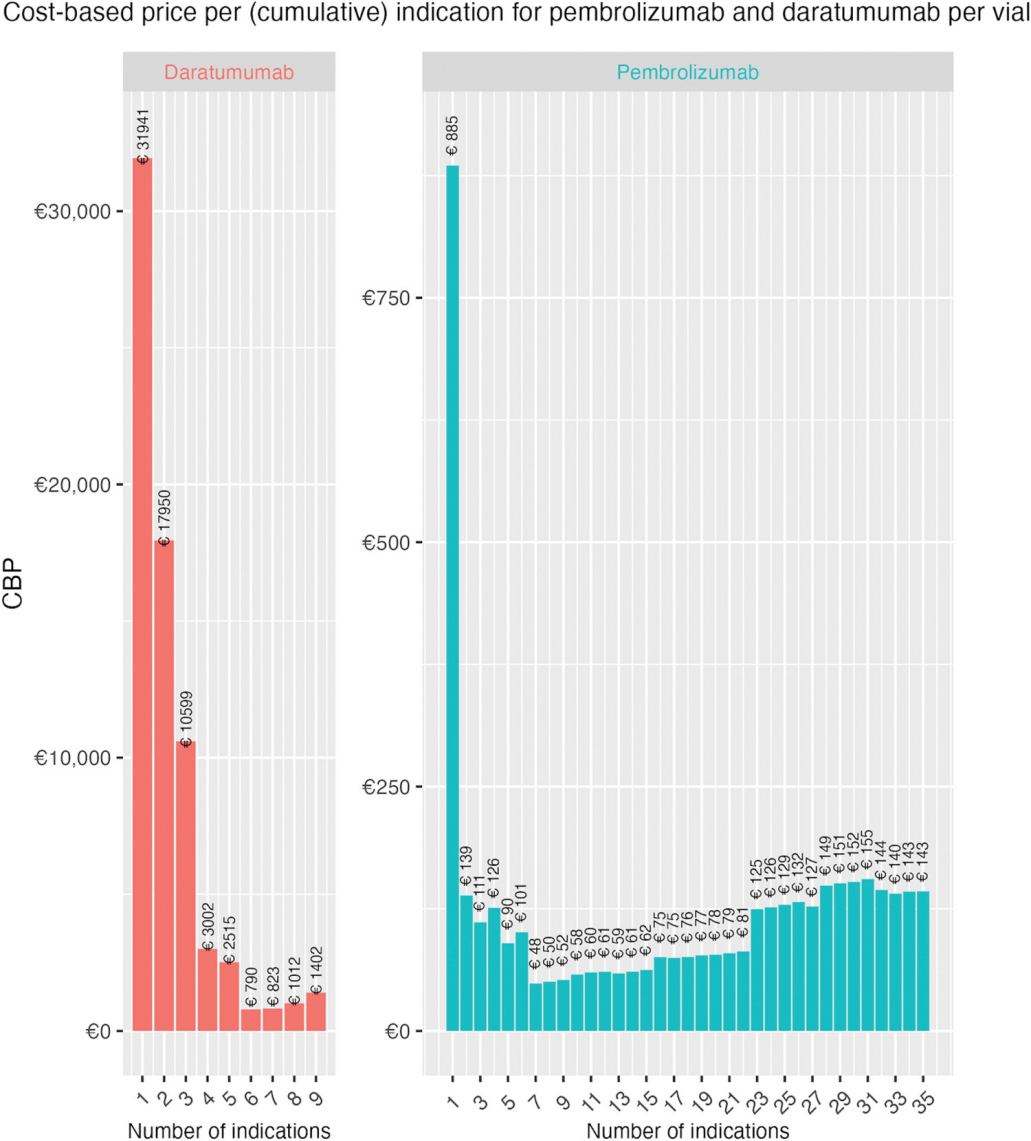
CBP per cumulative indication for daratumumab and pembrolizumab per vial.

The CBP algorithm was modified to enable indication-specific cumulative results, meaning that instead of being time-dependent in years (t) the algorithm is dependent on the number of indications (i). The algorithm sums R&D costs and eligible patients per new indication instead of per calendar year. Fig 2 depicts the estimated prices for each drug and cumulative indication. As–the first year after market access for both drugs only compromised one indication CBPs are identical.

The sharp decrease in price in the first few indications is consistent with large initial R&D costs and a small patient population in the first indications. The CBP per indication increases over time due to the shrinking remaining patent period and subsequent investments made in new indications. Obtained CBPs for pembrolizumab are in all cumulative indications lower than the actual list price in the United States (€5,350) and the Netherlands (€2,861). The difference ranges between -€1,976 –-€2,809 and -€4,465 –-€5,298 per vial for the Netherlands and United States, respectively (see appendix IX). CBPs of daratumumab fall both above and below the list prices in both the United States (€8,735) and the Netherlands (€4,766). The difference ranges between €23,2206 –€-7,945 and €27,175 –€-3,976 for the United States and the Netherlands, respectively (see appendix IX).

### Stepwise sensitivity analysis

Input parameters are most sensitive in the first years/indications and become less sensitive over time. The number of eligible patients was the most sensitive input, yielding a range between €662 –€45,581 per vial and €42 –€1,262 across years for both daratumumab and pembrolizumab, respectively. The larger variation in CBPs for daratumumab results from the small patient population in the first indication, namely MM patients that received at least three prior treatments. Varying the initial costs associated with R&D resulted in prices ranging between €676 –€41,489 for daratumumab and between €43 –€1,149 for pembrolizumab. The remaining two input parameters were to a smaller extent sensitive, ranging between €790 –€33,219 and €50 –€921 for daratumumab and pembrolizumab, respectively varying the profit margin and between €790 –€33,219 and €50 –€921 for manufacturing costs. Further results obtained within the stepwise DSA can be found in Appendix VII & VIII.

**Fig 3:**
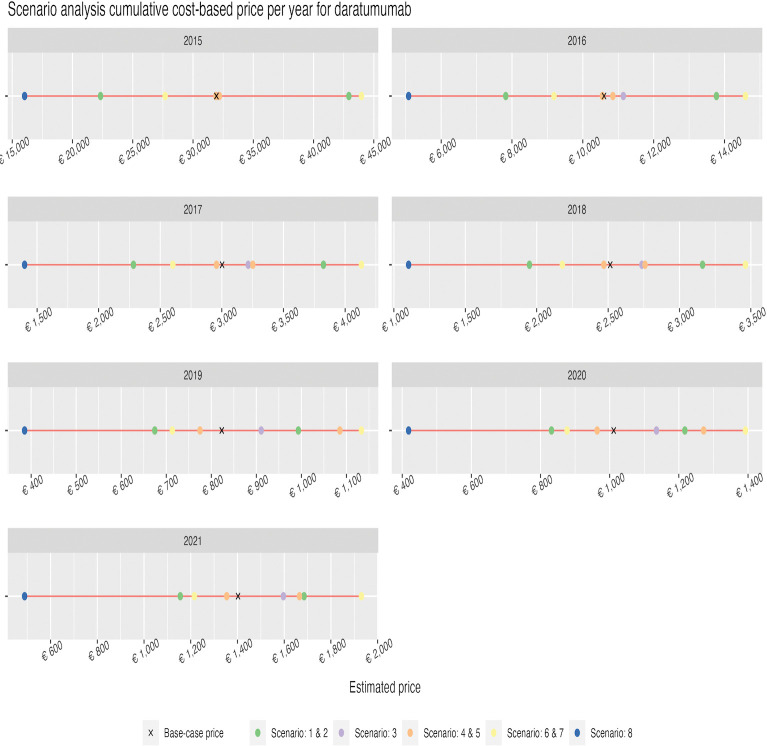
Scenario analysis for daratumumab using a cumulative cost-based price per year per vial.

**Fig 4:**
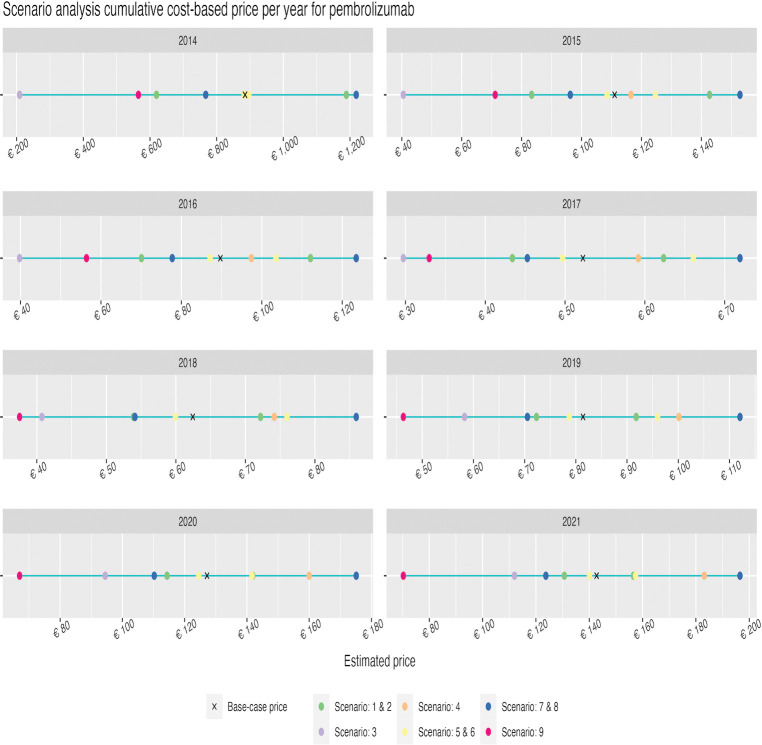
Scenario analysis for pembrolizumab using a cumulative cost-based price per year per vial.

Similarly to the DSA, the scenarios explored resulted in larger impacts in the earlier years/indications. The 8^th^ scenario, exploring a profit margin of 76.5%, resulted in the highest CBP for both daratumumab and pembrolizumab (i.e., €43,972 and €1219, respectively). The lowest estimates resulted from the 3^th^ scenario, implementing uncapitalized costs of R&D, namely €445 for daratumumab and €30 for pembrolizumab. Results from the scenario analysis can be found in Figs 3 and 4.

**Fig 5:**
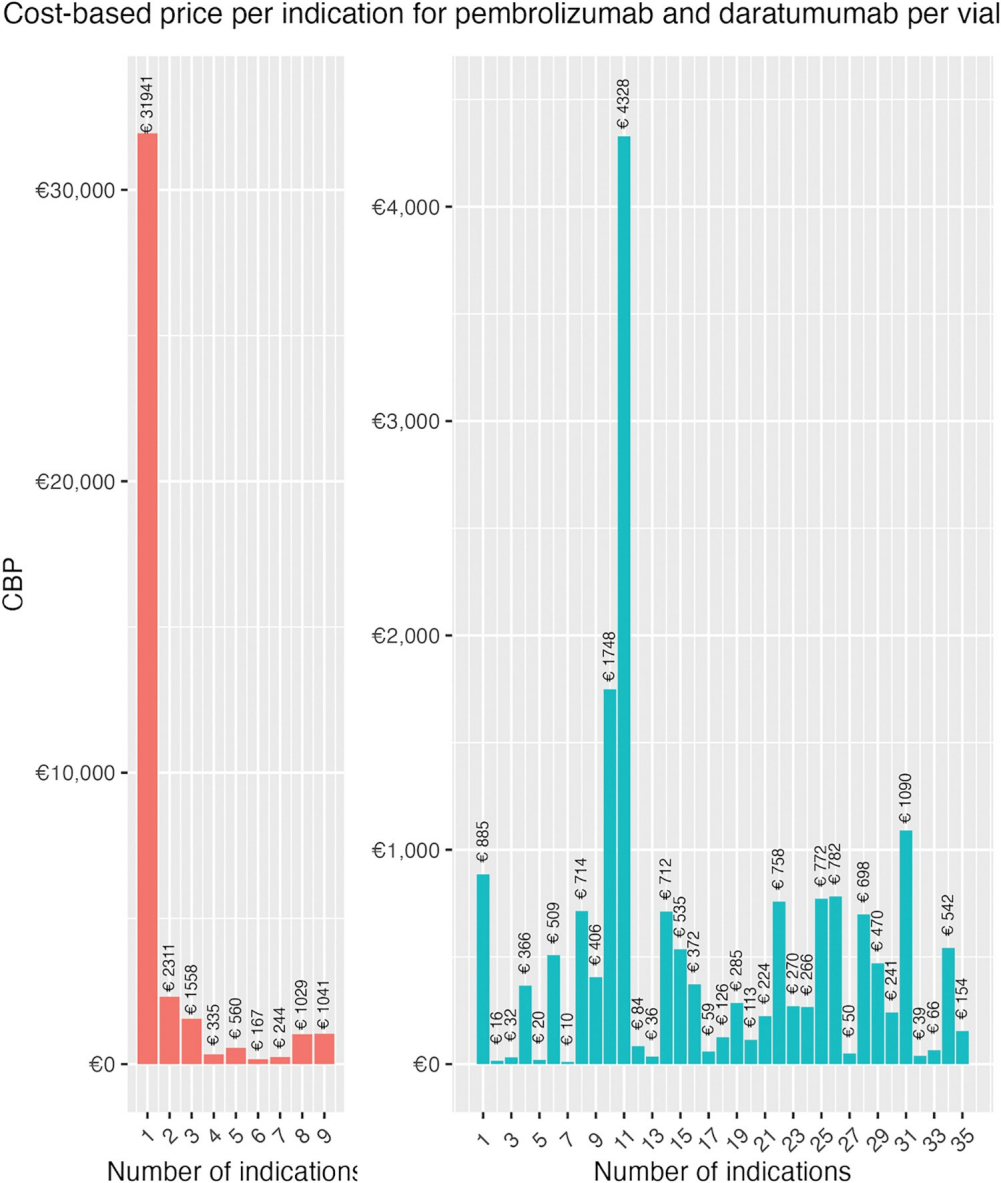
Implementation of indication-based pricing (10^th^ scenario) for daratumumab and pembrolizumab.

Instead of cumulative CPBs, the 10^th^ scenario explored indication-based pricing (IBP) (see Fig 5). IBP only considers the R&D spending made for a specific indication; therefore the first indication captures all initial R&D and later indications only the extra R&D spending. IBPs present higher variability due to the non-cumulative nature of the algorithm. IBCBPs ranged from €167 to €31,941 for daratumumab, showcasing considerably lower prices. Similarly, IBCBPs for pembrolizumab reached considerable lows, namely €16 per vial for NSCLC (2^nd^ indication). Low CBPs are attributable to larger patient populations and relatively small extra R&D costs. The highest CBP for pembrolizumab was calculated for the 11^th^ indication (refractory primary mediastinal large B-cell lymphoma), namely €4,328.

## Discussion

In this study, we investigated the application of a CBP model in pembrolizumab and daratumumab, two expensive oncology drugs with indication broadening. Obtained CBPs vary greatly over time or indications ranging between €823 –€31,941 and €52 –€885 for daratumumab and pembrolizumab, respectively. Modelled CBPs are foremost sensitive to the number of eligible patients and initial R&D costs.

Both daratumumab and pembrolizumab received EMA and FDA approval. Moreover, both drugs are approved in Australia by the Therapeutics Goods Administration (TGA) [36,37] and in Japan by the Pharmaceutical and Medicinal Devices Agency (PMDA) [38]. However, list prices differ across countries. List prices for daratumumab fall within our estimated CBPs depending on the year. In contrast, list prices for pembrolizumab were between 66.8% and 98.4% higher in the Netherlands than our estimated CBPs, mainly resulting from a large number of indications and, consequently, a high number of modelled eligible patients.

The approval for pembrolizumab took 188 days at the FDA and 408 days for the EMA [39]. After approval, patients did not always have access due to pricing and reimbursement (P&R) negotiations. In July 2017, the Netherlands reimbursed pembrolizumab for NSCLC and all upcoming indications, making a confidential price managed entry agreement (MEA) with Merck [40]. A report from the OECD (2019) found that 9 out of 14 of their members implemented MEAs for pembrolizumab [41]. In September 2018, a confidential price agreement was also enacted for daratumumab in the Netherlands [42]. Other countries i.e., Italy also have a MEA for daratumumab [43]. Since current P&R negotiations in the EU are predominantly grounded in value-based theory, where relative (cost-)effectiveness is assessed based on country specific criteria, differences amongst countries can lead to diverging decisions and ultimately disparate patient access.

### Strengths

The selection of daratumumab and pembrolizumab exemplifies the broadening of indications in histology-agnostic (i.e., pembrolizumab) and histology-specific (i.e., daratumumab) cancer treatments. The eligible patient population for each product was based on new incident cases. Although this potentially could have led to an underestimation of eligible patients due to the omission of prevalent cases, this is a conservative approach which would otherwise result in a lower CBP. The costs associated with initial R&D take into account the costs of failed trials, cost of capital (10.5%) and all clinical phases of the drug development, therefore, giving a realistic aggregate estimate of costs associated with the development of a drug. Moreover, the utilized estimates are specific for antineoplastic and immunomodulating agents, which is arguably more accurate than more general estimations.

To our knowledge, CBP models are not yet utilized for P&R negotiations. However, the use of CBP models in costly treatment P&R negotiations could facilitate curtailing excess profits. Moreover, the *“model’s flexibility”* enables payers to estimate a CBP for each year and/or indication, needing minimal extra inputs. If cost-based pricing facilitates lower list prices, price-sensitive prescribers might be less reluctant to prescribe these pharmaceuticals, resulting in broader patient access. Moreover, in countries where healthcare is publicly financed, lower drug prices could result in lower healthcare spending or create room for investments in other parts of the healthcare system.

### Limitations

The quality of the results obtained from the CBP models are dependent on the accurateness of inputs, therefore each model input needs scrutinizing.

First the costs associated with manufacturing of both pharmaceutical products are based on prices per gram of mAbs produced. These costs might, however, vary depending on the production process which was explored in the 5^th^ and 6^th^ scenarios. Moreover, the packaging and distribution of each product were omitted and therefore is likely to underestimate the actual costs. This is especially true for pembrolizumab since lower doses of active pharmaceutical ingredient (API) are packed per vial in comparison to daratumumab.

Secondly, initial R&D costs were extracted from Wouters et al. (2020) [17]. Although the distinction was made for antineoplastic and immunomodulating agents, the exact spending on R&D for each product could not be estimated. The use of one lumpsum has several drawbacks namely, both daratumumab and pembrolizumab received accelerated approval, reaching the market before phase III clinical trial results [44,45]. Moreover, daratumumab received orphan designation [46] and pembrolizumab received orphan designation for some indications [47] making both drugs eligible for tax credits. Government funded research or subsidies received are also not accounted for. Furthermore, double counting of additional R&D costs is possible since initial R&D costs were not dissected per indication, and therefore could potentially include R&D spending on additional indications, as acknowledged by Wouters et al. (2020) [17]. The R&D costs reported by Wouters et al. (2020) [17] range in the upper bound of estimates available in the literature [32]. Transparency on R&D costs per individual product is generally lacking in large pharmaceutical companies. The European Parliament adopted a resolution in November 2021 [48] that refers to the 72^nd^ World Health Assembly (WHA) resolution that took place in May 2019, where the importance of transparency of markets for medicines is stressed. Moreover, the 72^nd^ WHA resolution mentions the request to: “analyse the availability of data on inputs throughout the value chain, including data on clinical trials and price information”. If implemented, this could lead to greater transparency in R&D spending in the future. Improving transparency on R&D costs would increase the accuracy of the model estimates and contribute to a fair dialogue on pharmaceutical pricing.

Thirdly, more research is needed to accurately estimate the R&D costs associated with the broadening of indications. We used the price of repurposing a pharmaceutical product as a surrogate [18], however, this likely overestimates the cost of R&D since the development takes place within the same pharmaceutical company and likely in parallel resulting in better optimized processes. Per contra, the cost of failure in the indication broadening was not accounted for, since failure probabilities might not be similar to the failure rates in drug repurposing. However, the failure rate in drug repurposing is low (45%) compared to innovative drugs [49]. Non-profit repurposing has been successful in the past with smaller investments (i.e., Sanofi’s fexinidazole only required $55 million) [50]. Others estimated the cost of repurposing at ≥60% of the *de novo* drug discovery costs [51]. The International Association of Mutual Benefit Societies (AIM) developed a CBP algorithm that only uses 10% of the original costs of R&D for any subsequent indication.

In the CBP models the remaining patent period is fixed to the first indication. However, different indications can have divergent patent expiry times or market exclusivity expiry dates.

Another limitation of our model is the assumptions that revenue diminish to zero after patent expiration date. This results in a simplification of reality, it is possible–like in the case with adalimumab–that the originator keeps part of the market after patent expiration [52]. However, predicting the decay in revenue would have introduced more uncertainties in the models.

Lastly the population eligible is based on the MDR, these also include countries that may get delayed access or no access at all, i.e., Eastern European countries [39], this might lead to an overestimation of the eligible patient population especially in early years or indications. However, if there is any uptake outside MDR countries, the patient population might be underestimated, leading to an overestimation in CBPs.

It should be noted that our CBP algorithm estimates an ex-factory price excluding any value-added tax (VAT) or margins for wholesalers and thus not incorporates costs charged in a pharmacy. The price a pharmaceutical company charges is referred to as the ex-factory price.

If cost-based pricing would be implemented as a standalone evaluation system for P&R negotiations, this could discourage pharmaceutical companies to operate efficiently–since payers would pay for all R&D/operating costs–and could thus hinder the development of advanced new treatments. If cost-based pricing would be implemented as an additional requirement to Health Technology Assessment (HTA) procedures, a drawback could be possible delays in patient access, due to longer P&R negotiations, which could stifle innovation in the long-term, since investing in pharmaceutical sector becomes less attractive.

Moreover, cost-based pricing models do not integrate effectiveness and thus cannot inform policy makers which treatment choice would be preferable. Therefore cost-based pricing models might be complementary to value-based economic evaluation but cannot substitute currently used processes.

### Future possible improvements

The utilized CBP model currently lacks the ability to implement differential pricing amongst selected countries, namely MDR as defined by the UN. Differential pricing is entrenched in economic theory and commonly refers to *“positive price discrimination”*, where the price is varied across markets to accommodate for price-sensitive countries while maximizing profits [53]. A possibility to achieve a differential CBP could be to weigh the price based on the gross domestic product (GDP) per capita. However, implementation of differential pricing in CBP models must be studied further.

Secondly, the profit margin applied in the CBP model was fixed at 20 percent. A variable profit margin dependent on clinical benefit could be envisaged in the future. The profit margin for pharmaceuticals for cancers could, for example, be made dependent on the European Society for Medical Oncology Magnitude of Clinical Benefit Scale (ESMO-MCBS) [54].

## Implications for payers

The difference between list prices and calculated CBPs can vary greatly depending on the type of model and moment in time or the indication. However, the calculated CBP prices per year or cumulative indication for pembrolizumab always remained lower than United States and Dutch list prices (see appendix IX). This implies that if payers would–complementary to value-based pricing–introduce the need for CBP analysis, prices would negatively be affected. In the case of daratumumab the implications are rather different, namely due to small patient numbers in the first indications, CBP per cumulative indication surpass the United States and the Netherlands list prices considerably. We notice, however, a sharp decline over indications (and time) due to rising eligibility. Importantly prices must be renegotiated over time or with the introduction of new indications. In this instance, the use of CBP by payers could result in higher prices at first and drop in later indications.

## Recommendations

The implementation of a CBP model can be structured in various ways. we recommend making CBP models–if indication-based pricing is not applicable–dependent on a time interval (possibly 1 or 2 years), this enables price stability and reduces the need for continuous updates. The identification of broadening indications for pharmaceutical products should be considered for initial P&R negotiations and possible managed MEAs should be tailored accordingly. Precautions should be taken especially when implementing confidential price agreements with long lifespans or be flexible to allow periodic price adjustments.

## Conclusion

The implementation of a CBP model for pharmaceutical products which are subject to indication broadening i.e., pembrolizumab and daratumumab, shows the possibility for price reductions over time and/or indications. In countries that have IBPs i.e., France and Switzerland the IBCBP version of the algorithm could be utilized, aiding stakeholders in pricing of subsequent indications. In conclusion the use of CBP models can foster the dialogue on *fair pricing* for pharmaceutical products and can be remodelled to accommodate for indication broadening.

## Supporting information

S1 Appendix (DOCX)

S1 Data (XLSX)

S1 Fig (TIFF)

S2 Fig (TIFF)

S3 Fig (TIFF)

S4 Fig (TIFF)

S5 Fig (TIFF)

S6 Fig (TIFF)
