# Supplementary material for: Applying a cost-based pricing model for innovative cancer treatments subject to indication expansion: A case study for pembrolizumab and daratumumab
Source: PLoS One. 2024 Feb 1;19(2):e0293264. doi: 10.1371/journal.pone.0293264 (PMC10833582; doi:10.1371/journal.pone.0293264)
Supplement: S1 Appendix — (DOCX) [file pone.0293264.s001.docx]

## Appendix

Appendix I. All indications approved until 2022 for Pembrolizumab

| Indication | Year | Indication description | FDA Date | Trial FDA |
| --- | --- | --- | --- | --- |
| 1 | 2014 | Advanced or unresectable melanoma who are no longer responding to other drugs. | 04/09/2014 | KEYNOTE-001 |
| 2 | 2015 | Advanced (metastatic) non-small cell lung cancer (NSCLC) whose disease has progressed after other treatments and with tumors that express a protein called PD-L1. | 02/10/2015 | KEYNOTE-001 |
| 3 | 2015 | First-line treatment of patients with unresectable or metastatic melanoma. | 18/12/2015 | Phase 3 trial, KEYNOTE-006 |
| 4 | 2016 | Recurrent or metastatic head and neck squamous cell carcinoma (HNSCC) with disease progression on or after platinum-containing chemotherapy. | 05/08/2016 | KEYNOTE-012 |
| 5 | 2016 | First-line treatment of patients with metastatic non-small cell lung cancer (NSCLC) whose tumors have high PD-L1 expression (tumor proportion score [TPS] of 50 percent or more), with no EGFR or ALK genomic tumor aberrations. | 24/10/2016 | phase 3 KEYNOTE-024 |
| 6 | 2017 | Adult and pediatric patients with refractory classical Hodgkin lymphoma (cHL), or who have relapsed after three or more prior lines of therapy. | 14/03/2017 | KEYNOTE-087 |
| 7 | 2017 | Combination with Pemetrexed and Carboplatin, a commonly used chemotherapy regimen, for the first-line treatment of metastatic non-squamous NSCLC, irrespective of PD-L1 expression. | 10/05/2017 | KEYNOTE-021, Cohort G1 |
| 8 | 2017 | Advanced or metastatic urothelial carcinoma who are ineligible for cisplatin-containing chemotherapy. | 18/05/2017 | KEYNOTE-052 |
| 9 | 2017 | Previously Treated Patients with Recurrent Locally Advanced or Metastatic Gastric or Gastroesophageal Junction Cancer Whose Tumors Express PD-L1. | 22/09/2017 | KEYNOTE-059 |
| 10 | 2018 | Recurrent or metastatic cervical cancer with disease progression on or after chemotherapy whose tumors express PD-L1. | 12/06/2018 | KEYNOTE-158 Cohort E |
| 11 | 2018 | Adult and pediatric patients with refractory primary mediastinal large B-cell lymphoma (PMBCL), or who have relapsed after two or more prior lines of therapy. | 13/06/2018 | KEYNOTE-170 |
| 12 | 2018 | Combination with pemetrexed and platinum chemotherapy for the first-line treatment of patients with metastatic non-squamous non-small cell lung cancer (NSCLC), with no EGFR or ALK genomic tumor aberrations. | 20/08/2018 | Phase 3 KEYNOTE-189 |
| 13 | 2018 | Combination with carboplatin and either paclitaxel or nab-paclitaxel, for the first-line treatment of patients with metastatic squamous non-small cell lung cancer (NSCLC). | 30/10/2018 | Phase 3 KEYNOTE-407 |
| 14 | 2018 | Hepatocellular carcinoma (HCC) previously treated with sorafenib. | 09/11/2018 | KEYNOTE-224 |
| 15 | 2018 | Adult and pediatric patients with recurrent locally advanced or metastatic Merkel cell carcinoma (MCC). | 19/12/2018 | Phase 2 CITN-09/KEYNOTE-017 |
| 16 | 2019 | Adjuvant treatment of patients with melanoma with involvement of lymph node(s) following complete resection. | 19/02/2019 | Phase 3 EORTC1325/KEYNOTE-054 |
| 17 | 2019 | Monotherapy for the first-line treatment of patients with stage III non-small cell lung cancer (NSCLC) who are not candidates for surgical resection or definitive chemoradiation, or metastatic NSCLC, and whose tumors express PD-L1. | 11/04/2019 | Phase 3 KEYNOTE-042 |
| 18 | 2019 | Combination with Inlyta (axitinib), a tyrosine kinase inhibitor, for the first-line treatment of patients with advanced renal cell carcinoma (RCC). | 22/04/2019 | Phase 3 KEYNOTE-426 |
| 19 | 2019 | First-line treatment of patients with metastatic or with unresectable, recurrent head and neck squamous cell carcinoma (HNSCC). | 11/06/2019 | KEYNOTE-048 |
| 20 | 2019 | Monotherapy for the treatment of patients with metastatic small cell lung cancer (SCLC) with disease progression on or after platinum-based chemotherapy and at least one other prior line of therapy. | 18/06/2019 | KEYNOTE-158 (cohort G) and KEYNOTE-028 (cohort C1) |
| 21 | 2019 | Monotherapy for the treatment of patients with recurrent locally advanced or metastatic squamous cell carcinoma of the esophagus whose tumors express PD-L1. | 31/07/2019 | KEYNOTE-181 |
| 22 | 2019 | Combination with Lenvima for advanced endometrial carcinoma that is not microsatellite instability-high (MSI-H) or mismatch repair deficient (dMMR), patients with disease progression and not candidate for curative surgery or radiation. | 17/09/2019 | Phase 2 KEYNOTE-146/Study 111 |
| 23 | 2020 | Monotherapy for Bacillus Calmette-Guerin (BCG)-unresponsive, high-risk, non-muscle invasive bladder cancer (NMIBC) with carcinoma in situ (CIS) with or without papillary tumors who are ineligible for or have elected not to undergo cystectomy. | 08/01/2020 | KEYNOTE-057 |
| 24* | 2020 | Additional recommended dosage of 400 mg every six weeks (Q6W) for Keytruda, Merck’s anti-PD-1 therapy, across all adult indications, including monotherapy and combination therapy. | 28/04/2020 | na |
| 25* | 2020 | Monotherapy for the treatment of adult and pediatric patients with unresectable or metastatic tumor mutational burden-high (TMB-H) [≥10 mutations/megabase (mut/Mb)] solid tumors. | 17/06/2020 | KEYNOTE-158 |
| 26 | 2020 | Monotherapy for the treatment of patients with recurrent or metastatic cutaneous squamous cell carcinoma (cSCC) that is not curable by surgery or radiation. | 24/06/2020 | Phase 2 KEYNOTE-629 trial |
| 27 | 2020 | Monotherapy for the first-line treatment of patients with unresectable or metastatic microsatellite instability-high (MSI-H) or mismatch repair deficient (dMMR) colorectal cancer. | 29/06/2020 | KEYNOTE-177 |
| 28 | 2020 | Monotherapy for the treatment of adult patients with relapsed or refractory classical Hodgkin lymphoma (cHL). | 15/10/2020 | Phase 3 KEYNOTE-204 |
| 29 | 2020 | Combination with chemotherapy for the treatment of patients with locally recurrent unresectable or metastatic triple-negative breast cancer (TNBC) whose tumors express PD-L1 (Combined Positive Score [CPS] ≥10). | 13/11/2020 | Phase 3 KEYNOTE-355 trial |
| 30 | 2021 | Locally advanced or metastatic esophageal or gastroesophageal junction (GEJ), carcinoma that is not amenable to surgical resection or definitive chemoradiation in combination with platinum- and fluoropyrimidine-based chemotherapy. | 23/03/2021 | Phase 3 KEYNOTE-590 |
| 31 | 2021 | Combination with trastuzumab, fluoropyrimidine- and platinum-containing chemotherapy, for the first-line treatment of patients with locally advanced unresectable or metastatic HER2-positive gastric or gastroesophageal junction (GEJ) adenocarcinoma. | 05/05/2021 | Phase 3 KEYNOTE-811 trial |
| 32 | 2021 | Monotherapy for the treatment of patients with locally advanced cutaneous squamous cell carcinoma (cSCC) that is not curable by surgery or radiation. | 06/07/2021 | Phase 2 KEYNOTE-629 trial |
| 33 | 2021 | Combination with Lenvima, for advanced endometrial carcinoma that is not microsatellite instability-high (MSI-H) or mismatch repair deficient (dMMR), with disease progression and not candidate for curative surgery or radiation. | 22/07/2021 | Phase 3 KEYNOTE-775/Study 309 trial |
| 34 | 2021 | High-risk early-stage triple-negative breast cancer (TNBC) in combination with chemotherapy as neoadjuvant treatment and then continued as a single agent as adjuvant treatment after surgery. | 27/07/2021 | Phase 3 KEYNOTE-522 trial |
| 35 | 2021 | Combination with Lenvima, for the first-line treatment of adult patients with advanced renal cell carcinoma (RCC). | 11/08/2021 | Phase 3 CLEAR (Study 307)/KEYNOTE-581 trial |
| 36 | 2021 | Combination with chemotherapy, with or without bevacizumab, for the treatment of patients with persistent, recurrent or metastatic cervical cancer whose tumors express PD-L1 (Combined Positive Score [CPS] ≥1). | 13/10/2021 | Phase 3 KEYNOTE-826 |
| 37 | 2021 | Adjuvant treatment of patients with renal cell carcinoma (RCC) at intermediate-high or high risk of recurrence following nephrectomy or following nephrectomy and resection of metastatic lesions. | 18/11/2021 | Phase 3 KEYNOTE-564 trial |

*Indication not included in the CBP model.

Appendix II. All indications approved until 2022 for Daratumumab

| Indication | Year | Indication description | Date FDA | Trial FDA |
| --- | --- | --- | --- | --- |
| 1 | 2015 | Patients with multiple myeloma who have received at least three prior treatments. | 16/11/2015 | SIRIUS study & GEN501 study |
| 2 | 2016 | Patients with multiple myeloma who have received at least two prior treatments. | 20/05/2016** | GEN501 & MMY2002 & MMY1002 & MMY1001 & GEN503 |
| 3 | 2016 | Multiple myeloma patients combined with either lenalidomide/bortezomib + dexamethasone, one prior treatment. | 21/11/2016 | Phase 3 POLLUX & Phase 3 CASTOR |
| 4 | 2017 | Daratumumab + Pomalidomide and Dexamethasone for patients with multiple myeloma who have received at least two prior therapies (including lenalidomide and a proteasome inhibitor). | 16/06/2017 | Phase I (MMY1001, EQUULEUS) |
| 5 | 2018 | Daratumumab + Bortezomib + Melphalan + Prednisone, newly diagnosed multiple myeloma patient’s ineligible for transplant. | 07/05/2018 | Phase 3 ALCYONE |
| 6* | 2019 | Split-dosing regimen for Ddaratumumab, option to split the first infusion over two consecutive days. | 12/02/2019 | Phase 1b EQUULEUS |
| 7 | 2019 | Daratumumab + lenalidomide + dexamethasone, newly diagnosed transplant ineligible | 27/06/2019 | Phase 3 MAIA |
| 8 | 2019 | Daratumumab + Bortezomib + Thalidomide + Dexamethasone (VTd) for newly diagnosed patients with multiple myeloma who are eligible for ASCT. | 26/09/2019 | Phase 3 CASSIOPEIA |
| 9* | 2020 | Subcutaneous formulation Daratumumab. | 01/05/2020 | Phase 3 COLUMBA |
| 10 | 2020 | Daratumumab + Carfilzomib + Dexamethasone R/R multiple myeloma patients who received three previous lines of therapy. | 20/08/2020 | Phase 3 CANDOR trial |
| 11 | 2021 | Daratumumab + Pomalidomide + Dexamethasone for adult patients with multiple myeloma who received one prior therapy containing a proteasome inhibitor and lenalidomide and were lenalidomide-refractory. | 20/05/2021** | Phase 3 APOLLO |

*Indication not included in the CBP model.

**Date from EMA approval.

Appendix III. Inputs utilized to calculate the patient population for Pembrolizumab

| Indication | Incidence crude rate per 100,000 | Subtype cancer % | Stage | Percent treated | Patients not in clinical trial % | % PD-L1 expression | Percent expected | MS 2014 | MS 2015 | MS 2016 | MS 2017 | MS 2018 | MS 2019 | MS 2020 | MS 2021 |
| --- | --- | --- | --- | --- | --- | --- | --- | --- | --- | --- | --- | --- | --- | --- | --- |
| 1 | 21.7 | 1.00 | 0.23 | 0.75 | 0.90 | 1.00 | 0.21 | 0.50 | 0.33 | 0.33 | 0.33 | 0.33 | 0.33 | 0.33 | 0.33 |
| 2 | 69.5 | 0.84 | 0.81 | 0.75 | 0.90 | 0.55 | 1.00 | 0.00 | 0.50 | 0.50 | 0.50 | 0.50 | 0.50 | 0.50 | 0.50 |
| 3 | 21.7 | 1.00 | 0.23 | 0.75 | 0.90 | 1.00 | 1.00 | 0.00 | 0.33 | 0.33 | 0.33 | 0.33 | 0.33 | 0.33 | 0.33 |
| 4 | 20.76 | 1.00 | 0.37 | 0.75 | 0.90 | 1.00 | 0.08 | 0.00 | 0.00 | 0.50 | 0.50 | 0.50 | 0.50 | 0.50 | 0.50 |
| 5 | 69.5 | 0.84 | 0.81 | 0.75 | 0.90 | 0.55 | 1.00 | 0.00 | 0.00 | 0.50 | 0.50 | 0.50 | 0.50 | 0.50 | 0.50 |
| 6 | 2.5 | 1.00 | 1.00 | 0.75 | 0.90 | 1.00 | 0.09 | 0.00 | 0.00 | 0.00 | 1.00 | 1.00 | 1.00 | 0.50 | 0.50 |
| 7 | 69.5 | 0.84 | 0.81 | 0.75 | 0.90 | 1.00 | 1.00 | 0.00 | 0.00 | 0.00 | 1.00 | 1.00 | 1.00 | 0.50 | 0.50 |
| 8 | 26.3 | 1.00 | 0.15 | 0.75 | 0.90 | 0.50 | 1.00 | 0.00 | 0.00 | 0.00 | 0.50 | 0.50 | 0.50 | 0.50 | 0.50 |
| 9 | 32.1 | 1.00 | 0.29 | 0.67 | 0.90 | 1.00 | 0.15 | 0.00 | 0.00 | 0.00 | 1.00 | 1.00 | 1.00 | 1.00 | 0.50 |
| 10 | 13.3 | 1.00 | 1.00 | 0.75 | 0.90 | 1.00 | 0.03 | 0.00 | 0.00 | 0.00 | 0.00 | 1.00 | 1.00 | 1.00 | 1.00 |
| 11 | 19.2 | 0.03 | 0.15 | 0.75 | 0.90 | 1.00 | 1.00 | 0.00 | 0.00 | 0.00 | 0.00 | 1.00 | 1.00 | 1.00 | 1.00 |
| 12 | 69.5 | 0.84 | 0.28 | 0.60 | 0.90 | 1.00 | 0.18 | 0.00 | 0.00 | 0.00 | 0.00 | 1.00 | 1.00 | 0.50 | 0.50 |
| 13 | 69.5 | 0.84 | 0.21 | 0.60 | 0.90 | 1.00 | 0.70 | 0.00 | 0.00 | 0.00 | 0.00 | 1.00 | 1.00 | 0.50 | 0.50 |
| 14 | 14.4 | 0.75 | 0.09 | 0.75 | 0.90 | 1.00 | 1.00 | 0.00 | 0.00 | 0.00 | 0.00 | 0.50 | 0.50 | 0.33 | 0.33 |
| 15 | 80.3 | 1.00 | 0.00 | 0.75 | 0.90 | 1.00 | 1.00 | 0.00 | 0.00 | 0.00 | 0.00 | 1.00 | 1.00 | 1.00 | 1.00 |
| 16 | 21.7 | 1.00 | 0.10 | 0.75 | 0.90 | 1.00 | 0.57 | 0.00 | 0.00 | 0.00 | 0.00 | 0.00 | 0.50 | 0.50 | 0.50 |
| 17 | 69.5 | 0.84 | 0.81 | 0.75 | 0.90 | 0.55 | 0.40 | 0.00 | 0.00 | 0.00 | 0.00 | 0.00 | 0.50 | 0.50 | 0.50 |
| 18 | 19.3 | 0.77 | 0.42 | 0.75 | 0.90 | 1.00 | 0.50 | 0.00 | 0.00 | 0.00 | 0.00 | 0.00 | 0.33 | 0.33 | 0.33 |
| 19 | 20.76 | 1.00 | 0.49 | 0.50 | 0.90 | 1.00 | 0.44 | 0.00 | 0.00 | 0.00 | 0.00 | 0.00 | 1.00 | 1.00 | 1.00 |
| 20 | 69.5 | 0.16 | 0.67 | 0.75 | 0.90 | 1.00 | 0.54 | 0.00 | 0.00 | 0.00 | 0.00 | 0.00 | 1.00 | 0.50 | 0.50 |
| 21 | 8 | 1.00 | 1.00 | 0.75 | 0.90 | 0.50 | 1.00 | 0.00 | 0.00 | 0.00 | 0.00 | 0.00 | 1.00 | 1.00 | 0.50 |
| 22 | 33.7 | 1.00 | 0.01 | 0.75 | 0.90 | 1.00 | 1.00 | 0.00 | 0.00 | 0.00 | 0.00 | 0.00 | 1.00 | 1.00 | 1.00 |
| 23 | 26.3 | 1.00 | 0.29 | 0.75 | 0.90 | 0.50 | 0.50 | 0.00 | 0.00 | 0.00 | 0.00 | 0.00 | 0.00 | 1.00 | 1.00 |
| 24* | na | na | na | na | na | na | na | na | na | na | na | na | na | na | na |
| 25* | na | na | na | na | na | na | na | na | na | na | na | na | na | na | na |
| 26 | 80.3 | 0.22 | 0.04 | 0.75 | 0.90 | 1.00 | 1.00 | 0.00 | 0.00 | 0.00 | 0.00 | 0.00 | 0.00 | 1.00 | 1.00 |
| 27 | 68.1 | 1.00 | 0.15 | 0.75 | 0.90 | 1.00 | 0.05 | 0.00 | 0.00 | 0.00 | 0.00 | 0.00 | 0.00 | 0.33 | 0.33 |
| 28 | 2.5 | 0.95 | 1.00 | 0.75 | 0.90 | 1.00 | 0.09 | 0.00 | 0.00 | 0.00 | 0.00 | 0.00 | 0.00 | 1.00 | 1.00 |
| 29 | 142 | 0.12 | 0.30 | 0.75 | 0.90 | 1.00 | 1.00 | 0.00 | 0.00 | 0.00 | 0.00 | 0.00 | 0.00 | 1.00 | 1.00 |
| 30 | 8 | 1.00 | 1.00 | 0.75 | 0.90 | 1.00 | 0.14 | 0.00 | 0.00 | 0.00 | 0.00 | 0.00 | 0.00 | 0.00 | 0.50 |
| 31 | 8 | 1.00 | 0.25 | 0.75 | 0.90 | 0.50 | 1.00 | 0.00 | 0.00 | 0.00 | 0.00 | 0.00 | 0.00 | 0.00 | 0.50 |
| 32 | 80.3 | 0.22 | 0.05 | 0.75 | 0.90 | 1.00 | 1.00 | 0.00 | 0.00 | 0.00 | 0.00 | 0.00 | 0.00 | 0.00 | 1.00 |
| 33 | 33.7 | 1.00 | 0.01 | 0.75 | 0.90 | 1.00 | 1.00 | 0.00 | 0.00 | 0.00 | 0.00 | 0.00 | 0.00 | 0.00 | 1.00 |
| 34 | 142 | 0.12 | 0.60 | 0.75 | 0.90 | 1.00 | 1.00 | 0.00 | 0.00 | 0.00 | 0.00 | 0.00 | 0.00 | 0.00 | 1.00 |
| 35 | 19.3 | 0.90 | 0.36 | 0.75 | 0.90 | 1.00 | 0.56 | 0.00 | 0.00 | 0.00 | 0.00 | 0.00 | 0.00 | 0.00 | 0.50 |
| 36 | 13.3 | 1.00 | 1.00 | 0.75 | 0.90 | 1.00 | 0.03 | 0.00 | 0.00 | 0.00 | 0.00 | 0.00 | 0.00 | 0.00 | 1.00 |
| 37 | 19.3 | 0.90 | 0.36 | 0.75 | 0.90 | 1.00 | 0.46 | 0.00 | 0.00 | 0.00 | 0.00 | 0.00 | 0.00 | 0.00 | 0.50 |

* Indication not included in the CBP model.

Appendix IV. Inputs utilized to calculate the patient population for daratumumab.

| Indication | Incidence crude rate per 100,000 | Subtype cancer | Symptomatic | Percent treated | Patients not in clinical trial | Percent Line treatment | Percent expected | MS 2015 | MS 2016 | MS 2017 | MS 2018 | MS 2019 | MS 2020 | MS 2021 |
| --- | --- | --- | --- | --- | --- | --- | --- | --- | --- | --- | --- | --- | --- | --- |
| 1 | 7.6 | 1.00 | 0.90 | 1.00 | 0.90 | 0.15 | 0.13 | 1.00 | 1.00 | 1.00 | 1.00 | 1.00 | 1.00 | 1.00 |
| 2 | 7.6 | 1.00 | 0.90 | 1.00 | 0.90 | 0.38 | 0.07 | 0.00 | 1.00 | 1.00 | 1.00 | 1.00 | 1.00 | 1.00 |
| 3 | 7.6 | 1.00 | 0.90 | 1.00 | 0.90 | 0.61 | 0.02 | 0.00 | 1.00 | 1.00 | 1.00 | 1.00 | 1.00 | 1.00 |
| 4 | 7.6 | 1.00 | 0.90 | 1.00 | 0.90 | 0.61 | 0.45 | 0.00 | 0.00 | 1.00 | 1.00 | 1.00 | 1.00 | 1.00 |
| 5 | 7.6 | 1.00 | 0.90 | 1.00 | 0.90 | 1.00 | 0.07 | 0.00 | 0.00 | 0.00 | 1.00 | 1.00 | 1.00 | 1.00 |
| 6* | na | na | na | na | na | na | na | na | na | na | na | na | na | na |
| 7 | 7.6 | 1.00 | 0.90 | 1.00 | 0.90 | 1.00 | 0.43 | 0.00 | 0.00 | 0.00 | 0.00 | 1.00 | 1.00 | 1.00 |
| 8 | 7.6 | 1.00 | 0.90 | 0.95 | 0.90 | 1.00 | 0.48 | 0.00 | 0.00 | 0.00 | 0.00 | 1.00 | 1.00 | 1.00 |
| 9* | na | na | na | na | na | na | na | na | na | na | na | na | na | na |
| 10 | 7.6 | 1.00 | 0.90 | 1.00 | 0.90 | 0.15 | 0.48 | 0.00 | 0.00 | 0.00 | 0.00 | 0.00 | 1.00 | 1.00 |
| 11 | 7.6 | 1.00 | 0.90 | 1.00 | 0.90 | 0.38 | 0.48 | 0.00 | 0.00 | 0.00 | 0.00 | 0.00 | 0.00 | 1.00 |

Appendix V. Inputs to calculate dosage pembrolizumab

| Indication | Median PFS (months) | Confidence interval | Fixed mg dose | Mg per kg | Days between doses | Total dose mg |
| --- | --- | --- | --- | --- | --- | --- |
| 1 | 5.5 | 3.4 - 6.9 | 0 | 10 | 14 | 8968 |
| 2 | 10.3 | 6.7 - na | 200 | 0 | 21 | 2986 |
| 3 | 5.5 | 3.4 - 6.9 | 0 | 10 | 14 | 8968 |
| 4 | 2.1 | 1.9 - 2.1 | 0 | 10 | 14 | 3424 |
| 5 | 7.9 | na | 200 | 0 | 21 | 2290 |
| 6 | 13.6 | 11.1 - 16.7 | 200 | 0 | 21 | 3942 |
| 7 | 8.8 | 7.6 - 9.2 | 200 | 0 | 21 | 2551 |
| 8 | 2.2 | na | 200 | 0 | 21 | 638 |
| 9 | 3.0 | na | 200 | 0 | 21 | 870 |
| 10 | 2.1 | na | 200 | 0 | 21 | 609 |
| 11 | 4.3 | 2.8 - 13.8 | 200 | 0 | 21 | 1246 |
| 12 | 9.0 | 8.1 - 9.9 | 200 | 0 | 21 | 2609 |
| 13 | 8.0 | 6.3 - 8.4 | 200 | 0 | 21 | 2319 |
| 14 | 4.9 | 3.5 -6.7 | 200 | 0 | 21 | 1420 |
| 15 | 16.8 | 4.6 - 43.4 | 0 | 2 | 21 | 3652 |
| 16 | 8.5 | 5.7 - 15.2 | 200 | 0 | 21 | 2464 |
| 17 | 7.1 | na | 200 | 0 | 21 | 2058 |
| 18 | 15.7 | 13.6 - 20.2 | 200 | 0 | 21 | 4551 |
| 19 | 2.3 | na | 200 | 0 | 21 | 667 |
| 20 | 4.5 | 4.3 - 5.4 | 200 | 0 | 21 | 1304 |
| 21 | 2.2 | 2.1 - 3.2 | 200 | 0 | 21 | 638 |
| 22 | 7.4 | 5.3 - 8.7 | 200 | 0 | 21 | 2145 |
| 23 | 4.2 | 3.4 - 9.1 | 200 | 0 | 21 | 1217 |
| 24* | na | na | na | na | na | na |
| 25* | na | na | na | na | na | na |
| 26 | 12.0 | na | 200 | 0 | 21 | 3478 |
| 27 | 16.5 | na | 200 | 0 | 21 | 4783 |
| 28 | 13.2 | na | 200 | 0 | 21 | 3826 |
| 29 | 9.7 | na | 200 | 0 | 21 | 2812 |
| 30 | 6.3 | 6.2 - 6.9 | 200 | 0 | 21 | 1826 |
| 31 | 10.6 | na | 200 | 0 | 21 | 3073 |
| 32 | 12.0 | na | 200 | 0 | 21 | 3478 |
| 33 | 6.6 | na | 200 | 0 | 21 | 1913 |
| 34 | 7.5 | na | 200 | 0 | 21 | 2174 |
| 35 | 23.9 | 20.8 - 27.7 | 200 | 0 | 21 | 6928 |
| 36 | 10.4 | 9.1 - 12.1 | 200 | 0 | 21 | 3015 |
| 37 | 11.8 | na | 200 | 0 | 21 | 3412 |

* Indication not included in the CBP model.

Appendix VI. Inputs to calculate dosage daratumumab

| Indication | Median PFS | Confidence interval | Mg per kg | Last dose schedule | Dose schedule I | Dose schedule II | Dose schedule III | Total dose mg |
| --- | --- | --- | --- | --- | --- | --- | --- | --- |
| 1 | 4.0 | 2.8 - 5.6 | 16 | 7 | na | na | na | 20871 |
| 2 | 3.7 | 2.76 - 4.63 | 16 | 7 | na | na | na | 19045 |
| 3 | 44.5 | 34.1 - na | 16 | 28 | 7 | 14 | 28 | 66208 |
| 4 | 8.8 | 4.6 - 15.4 | 16 | 28 | 7 | 14 | 28 | 19640 |
| 5 | 36.4 | 32.1 - 45.9 | 16 | 28 | 7 | 21 | 28 | 42084 |
| 6* | na | na | na | na | na | na | na | na |
| 7 | 60.0 | 54.8 - na | 16 | 28 | 7 | 14 | 28 | 86235 |
| 8 | 24.0 | na | 16 | 56 | 7 | 14 | 56 | 30053 |
| 9* | na | na | na | na | na | na | na | na |
| 10 | 28.6 | 22.7 - na | 16 | 28 | 7 | 14 | 28 | 30123 |
| 11 | 16.9 | na | 16 | 28 | 7 | 14 | 28 | 14861 |

* Indication not included in the CBP model.

Appendix VII. DSA analysis varying the profit margin and costs associated with manufacturing.

Appendix VIII. DSA analysis varying number of eligible patients and the initial cost of R&D

Appendix IX.
