# Supplementary figures and images for: Applying a cost-based pricing model for innovative cancer treatments subject to indication expansion: A case study for pembrolizumab and daratumumab
Source: PLoS One. 2024 Feb 1;19(2):e0293264. doi: 10.1371/journal.pone.0293264 (PMC10833582; doi:10.1371/journal.pone.0293264)

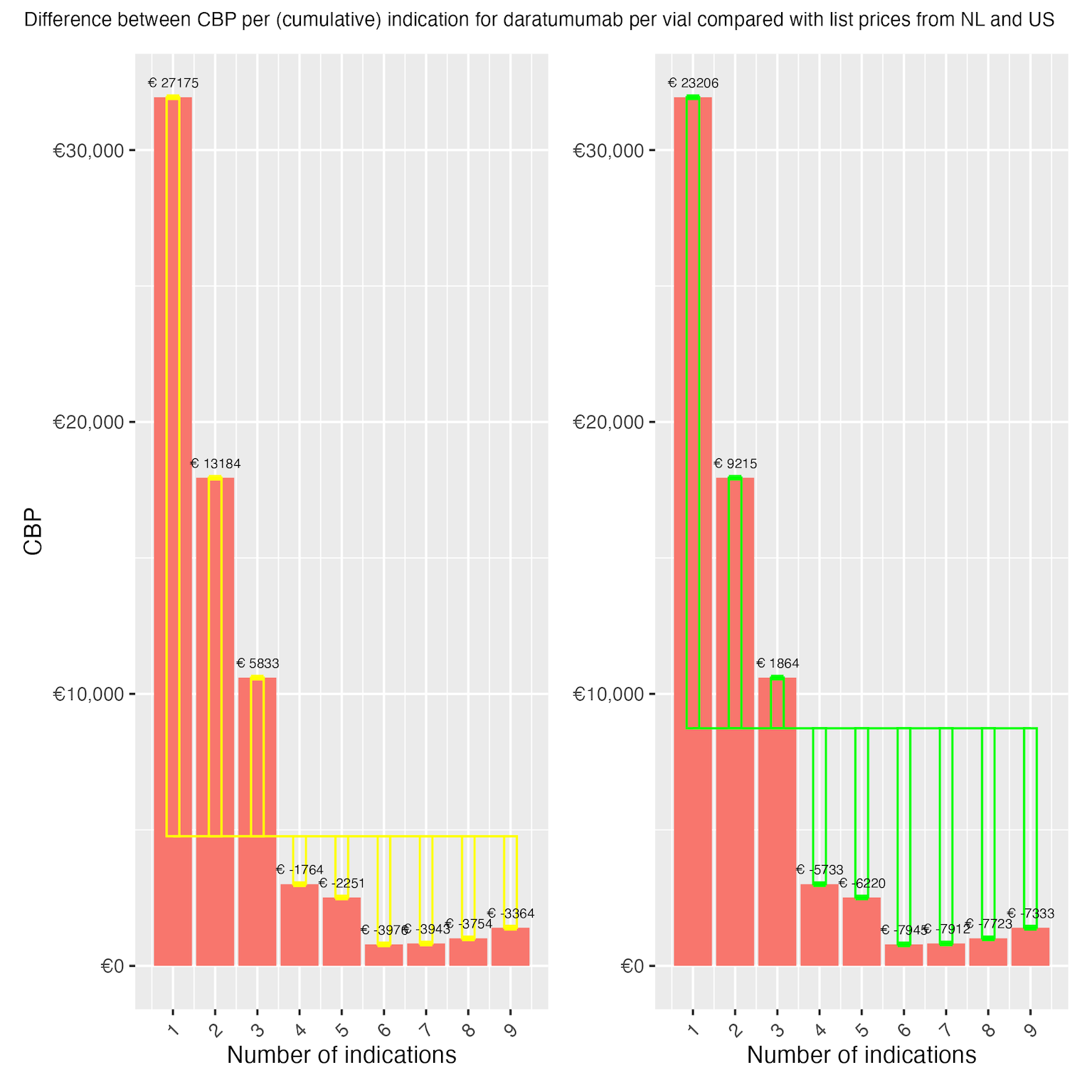

Supplement: S1 Fig — (TIFF) [file pone.0293264.s003.tiff]

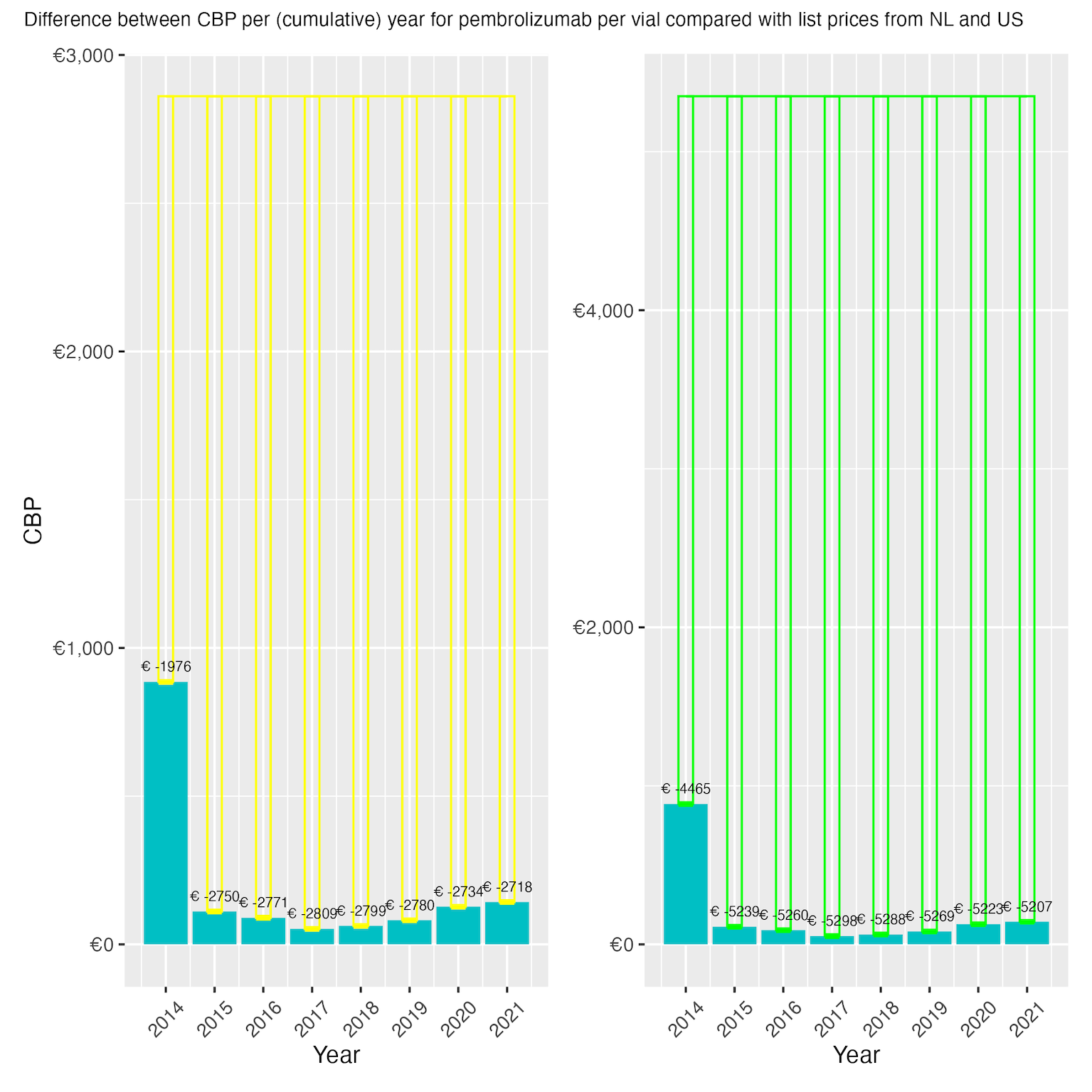

Supplement: S2 Fig — (TIFF) [file pone.0293264.s004.tiff]

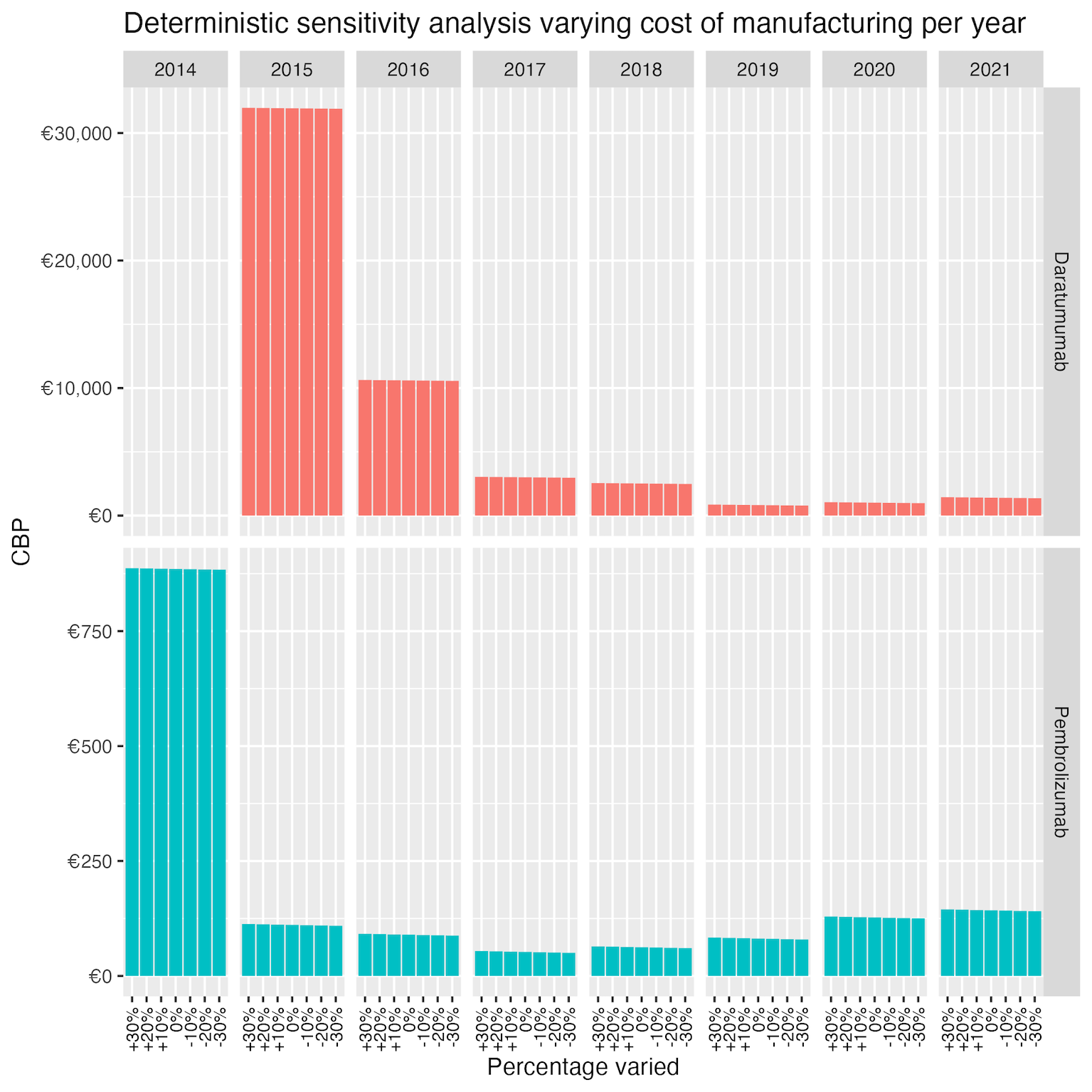

Supplement: S3 Fig — (TIFF) [file pone.0293264.s005.tiff]

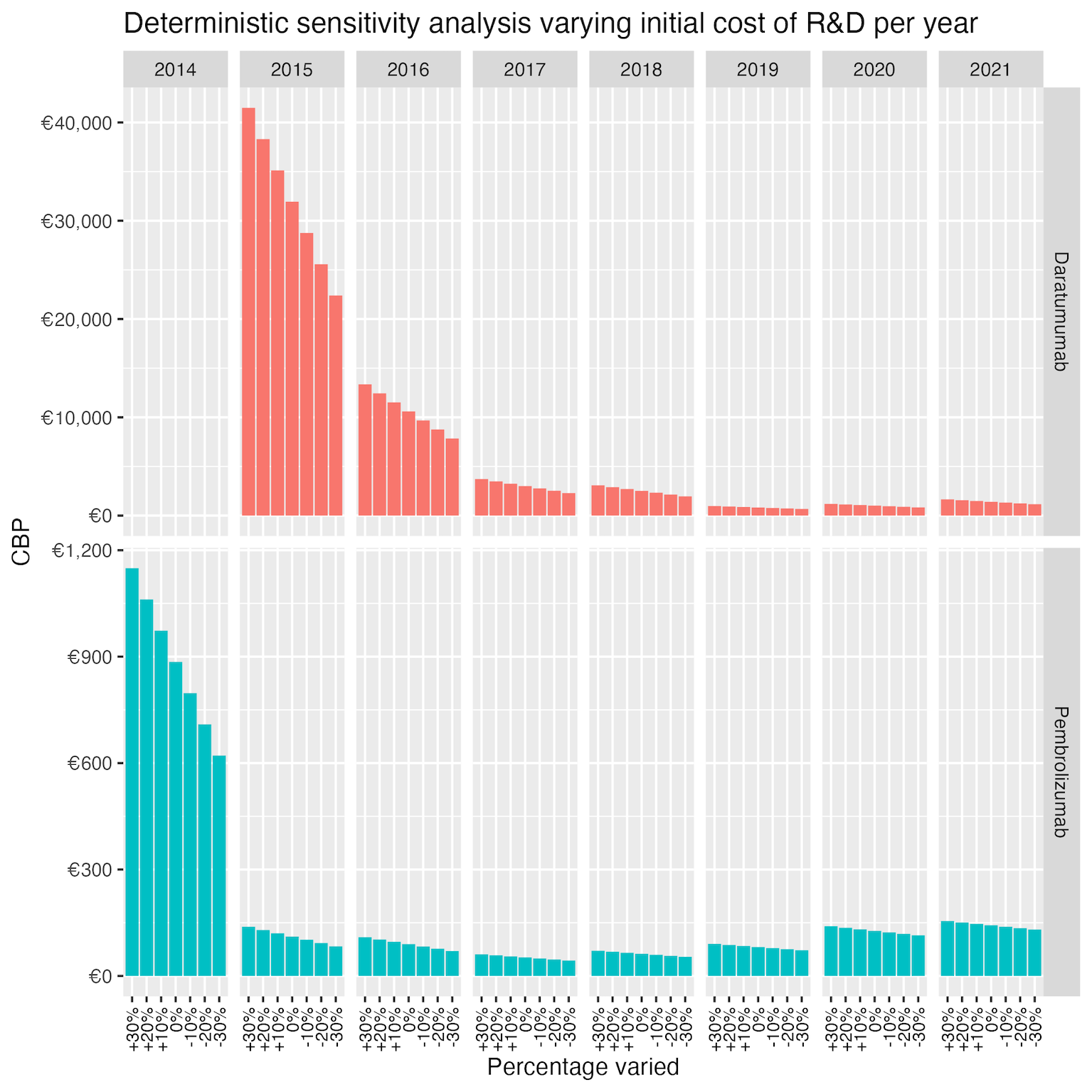

Supplement: S4 Fig — (TIFF) [file pone.0293264.s006.tiff]

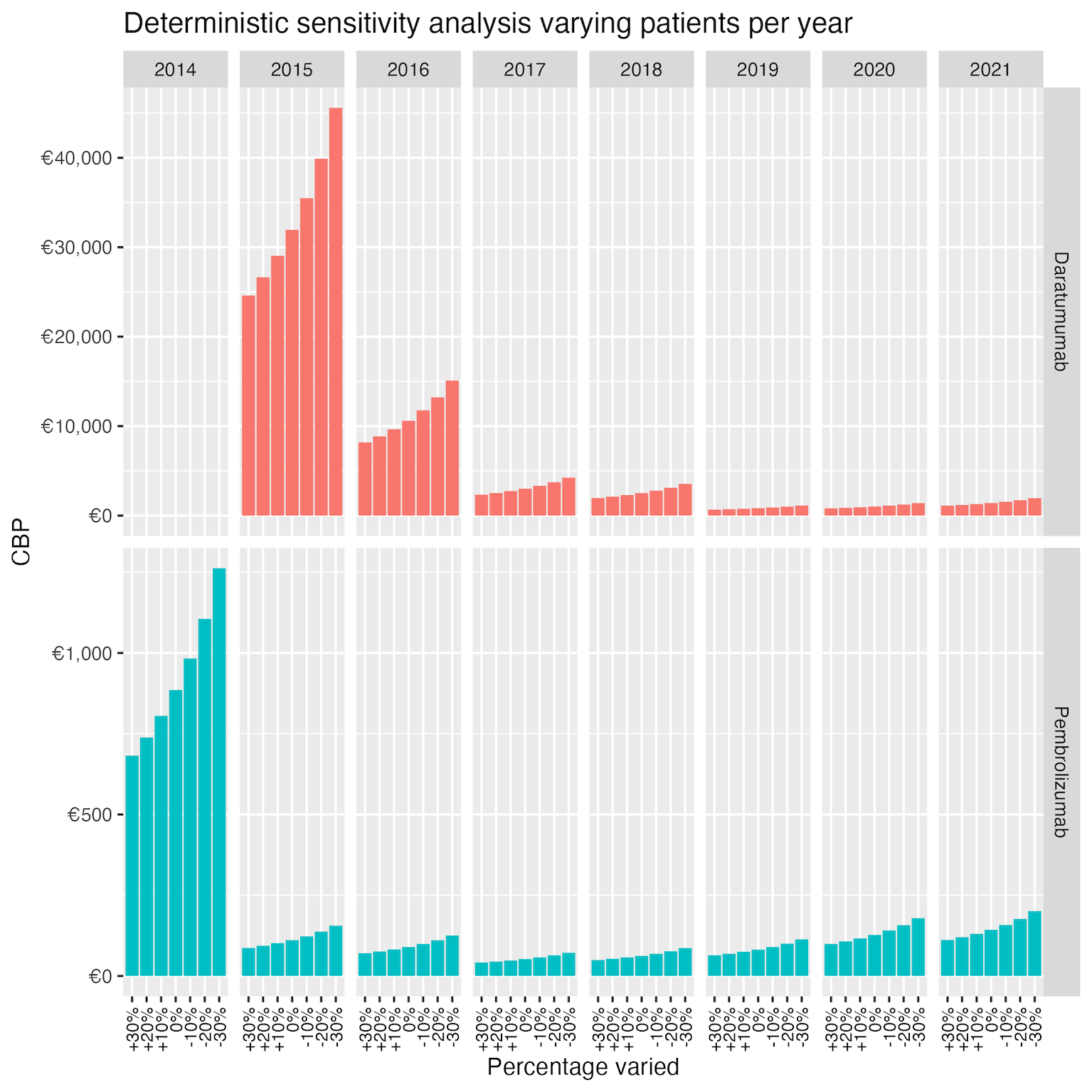

Supplement: S5 Fig — (TIFF) [file pone.0293264.s007.tiff]

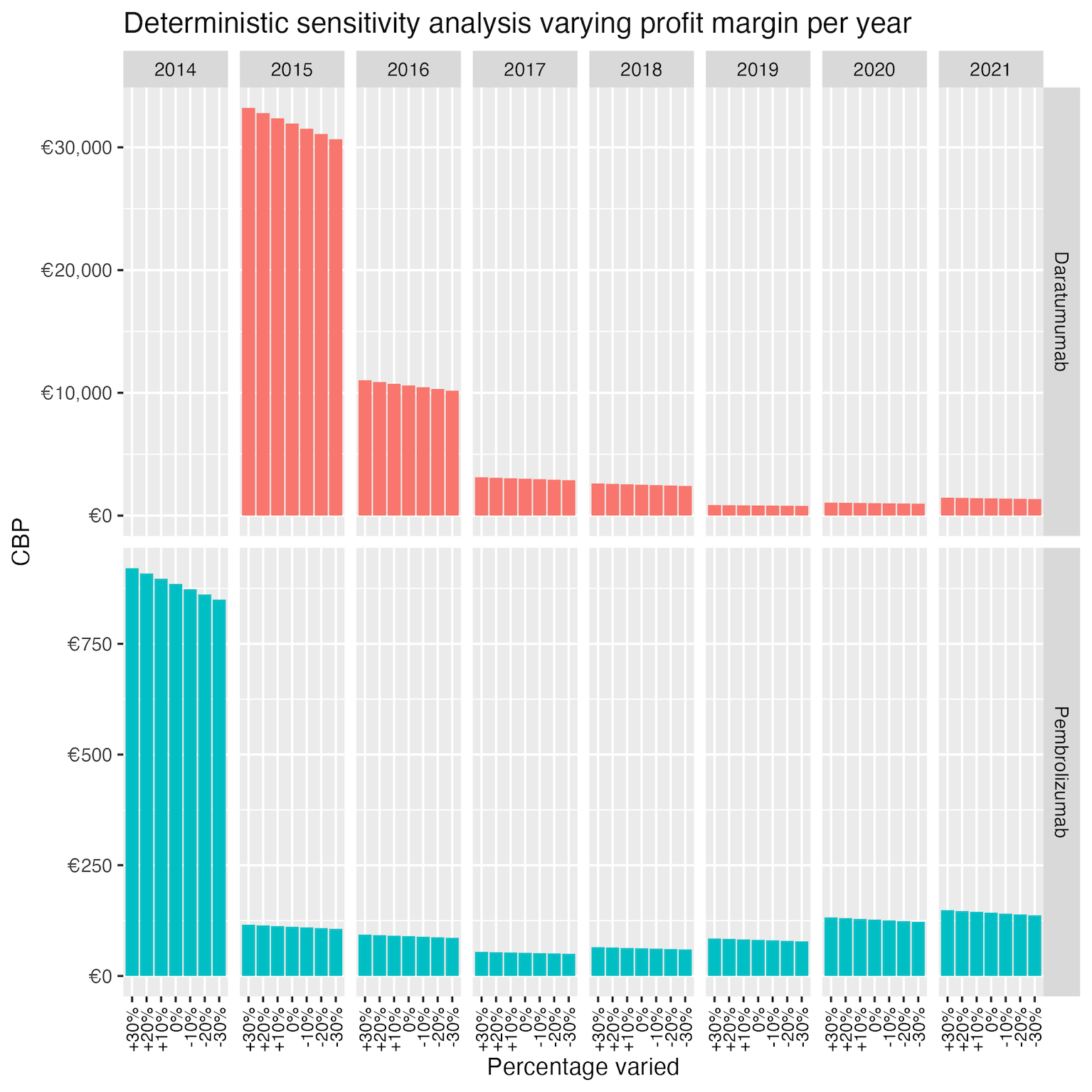

Supplement: S6 Fig — (TIFF) [file pone.0293264.s008.tiff]
